# SABO-ILSTSVR: a genomic prediction method based on improved least squares twin support vector regression

**DOI:** 10.3389/fgene.2024.1415249

**Published:** 2024-06-14

**Authors:** Rui Li, Jing Gao, Ganghui Zhou, Dongshi Zuo, Yao Sun

**Affiliations:** ^1^ College of Computer and Information Engineering, Inner Mongolia Agricultual University, Hohhot, China; ^2^ Inner Mongolia Autonomous Region Key Laboratory of Big Data Research and Application for Agriculture and Animal Husbandry, Hohhot, China; ^3^ Inner Mongolia Autonomous Region Big Data Center, Hohhot, China

**Keywords:** genomic prediction, LSTSVR, LASSO regularization, subtraction average based optimizer, high-dimensional data

## Abstract

In modern breeding practices, genomic prediction (GP) uses high-density single nucleotide polymorphisms (SNPs) markers to predict genomic estimated breeding values (GEBVs) for crucial phenotypes, thereby speeding up selection breeding process and shortening generation intervals. However, due to the characteristic of genotype data typically having far fewer sample numbers than SNPs markers, overfitting commonly arise during model training. To address this, the present study builds upon the Least Squares Twin Support Vector Regression (LSTSVR) model by incorporating a Lasso regularization term named ILSTSVR. Because of the complexity of parameter tuning for different datasets, subtraction average based optimizer (SABO) is further introduced to optimize ILSTSVR, and then obtain the GP model named SABO-ILSTSVR. Experiments conducted on four different crop datasets demonstrate that SABO-ILSTSVR outperforms or is equivalent in efficiency to widely-used genomic prediction methods. Source codes and data are available at: https://github.com/MLBreeding/SABO-ILSTSVR.

## 1 Introduction

With the decreasing cost of high-throughput sequencing data, genomic prediction (GP) emerges as a novel breeding approach, using high-density single nucleotide polymorphisms (SNPs) to capture associations between markers and phenotypes, thereby enabling prediction of genomic estimated breeding values (GEBVs) at an early stage of breeding (Meuwissen et al., 2001). Compared with conventional breeding methods, such as phenotype and marker-assisted selection, GP greatly shortens generation intervals, reduces costs, and enhances the efficiency and accuracy of new variety selection (Heffner et al., 2010).

From the proposal of the concept of genomic prediction to the present, a multitude of models have emerged. Early models primarily focused on improving best linear unbiased prediction (BLUP), such as ridge regression-based best linear unbiased prediction (rrBLUP) (Henderson, 1975) and genomic best linear unbiased prediction (GBLUP) (VanRaden, 2008), etc. In addition, researchers have proposed various Bayesian methods, including BayesA and BayesB (Meuwissen et al., 2001), BayesC (Habier et al., 2011) and BayesLasso (Park and Casella, 2008), Bayesian ridge regression (BayesRR) (da Silva et al., 2021), BSLMM(Zhou et al., 2013). Moreover, bayesian methods generally exhibit higher prediction accuracy than GBLUP in the majority of cases (Rolf et al., 2015). However, the Markov Chain Monte Carlo (MCMC) steps involved in parameter estimation for Bayesian methods can significantly increase computational costs. With advancements in high-throughput sequencing technologies, the increasing dimensionality of genotype data poses new challenges for GP models. To address this problem, some researchers have begun employing regularization term to mitigate the overfitting problem, such as ridge regression (Ogutu et al., 2012), Lasso (Usai et al., 2009), elastic net (Wang et al., 2019). Meanwhile, machine learning (ML) methods such as support vector regression (SVR) (Maenhout et al., 2007; Ogutu et al., 2011), random forest (RF) (Svetnik et al., 2003), gradient boosting decision tree (GBDT), extreme gradient boosting (XGBoost) (Chen and Guestrin, 2016) and light gradient boosting machine (LightGBM) (Ke et al., 2017), have made great performance in genomic prediction methods. With the development of deep learning (DL), researchers have also combined it with genomic prediction models, such as DeepGS proposed by Ma et al. (Ma et al., 2018), based on convolutional neural networks (CNN), and DNNGP proposed by Wang et al. (Kelin et al., 2023) for application in multi-omics, which have achieved better performance compared with other classic models. However, genotype data for most species exhibit high-dimensional small-sample characteristics, leading models often to fail to learn effective features from the training data. Moreover, most GP models contain a large number of parameters and there are significant differences in genotype data among different species, leading to a tedious parameter tuning process for each species, significantly increasing breeding costs. Therefore, enhancing the prediction performance of GP models and reducing the complexity of parameters tuning is of crucial importance for shortening generation intervals and reducing breeding costs.

This study explores a machine learning model, least squares twin support vector regression (LSTSVR), to address above problem. LSTSVR, proposed by Zhong et al. (2012), is a regression model that integrates the ideas of least squares method and twin support vector machine (TSVM) (Jayadeva et al., 2007). LSTSVR contains a kernel function; when linearly inseparable data exists in the original input space, it can become linearly separable after being mapped into a higher-dimensional feature space through an appropriate kernel function (Kung, 2014). LSTSVR improves the computational efficiency during the training process of traditional SVR models by introducing the least squares paradigm to replace the ε-insensitive loss function in SVR, thereby transforming the originally nonlinear optimization problem into an easier-to-solve system of linear equations, offering more stable performance. Simultaneously, adopting a two sets of support vectors for regression enhances the model’s learning capability and robustness (Huang et al., 2013). However, as a general-purpose regression model, LSTSVR still has certain limitations when dealing with various high-dimensional, small-sample genotype datasets.

To better address model overfitting and complex parameter tuning when the number of genotype samples is far less than the number of SNPs markers (Crossa et al., 2017; Tong and Nikoloski, 2020) this study has made improvements and optimizations to LSTSVR. Firstly, inspired by Lasso regularization, a penalty term was introduced to constrain model complexity on LSTSVR. Concurrently, in order to reduce the complexity stemming from model parameter tuning, the subtraction average-based optimizer (SABO) (Trojovský and Dehghani, 2023) was adopted to perform parameter optimization on the LSTSVR model. By combining the Lasso regularization-based ILSTSVR with the efficient optimization of SABO, this study successfully developed a genomic prediction model named SABO-ILSTSVR. To validate the effectiveness of SABO-ILSTSVR, comparative experiments were conducted using SABO-ILSTSVR on four different species datasets (maize, potato, wheat, and brassica napus) against commonly used genomic prediction models (LightGBM, rrBLUP, GBLUP, BSLMM, BayesRR, Lasso, RF, SVR, DNNGP). The results demonstrate that SABO-ILSTSVR exhibits equivalent or superior performance compared with widely-used genomic prediction methods. Finally, in order to reduce the difficulty of using the model, this study provides an easy-to-use python-based tool for breeders to use conveniently.

## 2 Materials and methods

### 2.1 Dataset

Four different crops datasets are used in this study, including potatoes, wheat, maize, and Brassica napus. The following provides detailed descriptions of the genotype and phenotype data for each dataset.

Potato dataset (Selga et al., 2021) is derived from a total of 256 cultivated varieties across three locations in northern and southern Sweden. Over 2000 SNPs markers used for genome-wide prediction were obtained from germplasm resources at both the Centro Internacional de la Papa (CIP, Lima, Peru) and those in the United States. According to Selga et al. (2021), this number of SNPs is already sufficient for predict genomic estimated breeding values (GEBVs) without loss of information. In this dataset, the total weight of tubers serves as the phenotype data.

Wheat dataset (Crossa et al., 2014) originates from the Global Wheat Program at CIMMYT, comprising information on 599 wheat lines. The project carried out numerous experiments across various environmental settings, with the dataset divided into four core environments according to distinct environmental parameters. Average grain yield (GY) serves as the phenotypic trait data within this dataset. It contains 1,279 SNPs markers, which were acquired following the removal of those with minor allele frequencies below 0.05 and the estimation of missing genotypes utilizing samples from the genotype edge distribution.

Maize dataset (Crossa et al., 2014) originates from CIMMYT’s maize project, comprising 242 maize lines and 46,374 SNP markers. The project encompasses multiple phenotypic data points, and we use the most significant yield-related traits as our phenotype data for this study.

Brassica napus dataset (Kole et al., 2002) is part of the MTGS package. The dataset comprises 50 lines derived from two varieties, 100 SNP markers, and phenotype information on flowering days across three distinct time periods (flower0, flower4, flower8).

### 2.2 SABO-ILSTSVR model

**FIGURE 1:**
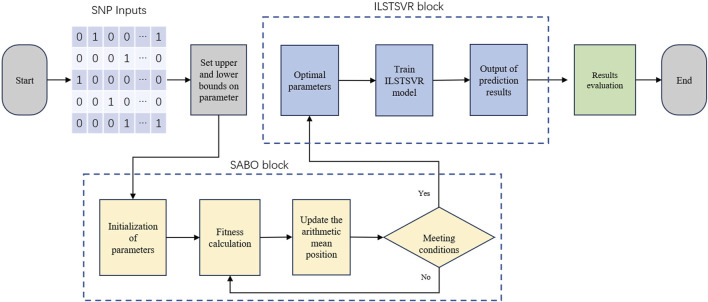
Illustration of SABO-ILSTSVR model frameworks.

The SABO-ILSTSVR model integrates the subtraction-based average optimizer (SABO) and an improved LSTSVR method. Its overall framework is depicted in Figure 1, followed by an elaboration of the model.

#### 2.2.1 LSTSVR

Least squares twin support vector regression (LSTSVR) is an improved twin support vector regression (TSVR) (Peng, 2010; Shao et al., 2013) model by Yang et al. (Lu Zhenxing, 2014). TSVR is an extension of the regression model derived from twin support vector machine (TSVM) (Jayadeva et al., 2007), suitable for addressing continuous value prediction problems, which accomplishes this by determining two regression functions, shown as follows,

$$f_{1}\left(X\right)=w_{1}^{T}X+b_{1},f_{2}\left(X\right)=w_{2}^{T}X+b_{2}$$ (1)

where Eq. (1) determines ε-insensitive down-bound function $f_{1}$ and ε-insensitive up-bound function $f_{2}$ , $w_{1}$ , $w_{2}$ are weight vectors, $b_{1}$ , $b_{2}$ are bias term. These can be obtained by solving the following quadratic programming problems (QPPs),

$$\begin{array}{c} \min_{w_{1},b_{1}}\;\frac{1}{2}{\left\|Y-e\varepsilon_{1}-\left(Xw_{1}+eb_{1}\right)\right\|}_{2}^{2}+C_{1}e^{T}\xi_{1}\\ s.t.\;Y-\left(Xw_{1}+eb_{1}\right)\geq -e\varepsilon_{1}-\xi_{1},\xi_{1}\geq 0, \end{array}$$ (2)

$$\begin{array}{c} \min_{w_{2},b_{2}}\;\frac{1}{2}{\left\|Y+e\varepsilon_{2}-\left(Xw_{2}+eb_{2}\right)\right\|}_{2}^{2}+C_{2}e^{T}\xi_{2}\\ s.t.\;-Y+\left(Xw_{2}+eb_{2}\right)\geq -e\varepsilon_{2}-\xi_{2},\xi_{2}\geq 0, \end{array}$$ (3)

where $C_{1}$ , $C_{2}$ are the positive penalty parameters, $\varepsilon_{1}$ , $\varepsilon_{2}$ are up- and down-bound parameters, $\xi_{1},\xi_{2}$ are slack vectors, e is a vector of ones with appropriate dimensions. The result of the final regression function is decided by the mean of upper and lower bound functions, as Eq. (4),

$$f\left(X\right)=\frac{1}{2}\left(f_{1}\left(X\right)+f_{2}\left(X\right)\right)=\frac{1}{2}\left(w_{1}^{T}+w_{2}^{T}\right)X+\frac{1}{2}\left(b_{l}+b_{2}\right)$$ (4)

In the spirit of LSTSVM, Yang et al. apply least squares method to TSVR. TSVR finds the optimal weight vector and bias terms by solving two QPPs, whereas LSTSVR transforms the original TSVR problem into two systems of linear equations for solution, which is typically faster and more stable than directly solving q QPPs, with the loss in accuracy being within an acceptable range. For LSTSVR model, the inequality constraints of (2) and (3) are replaced with equality constraints as follows,

$$\begin{array}{c} \min_{w_{1},b_{1}}\;\frac{1}{2}{\left\|Y-e\varepsilon_{1}-\left(Xw_{1}+eb_{1}\right)\right\|}_{2}^{2}+C_{1}{\xi_{1}}^{T}\xi_{1}\\ s.t.\;Y-\left(Xw_{1}+eb_{1}\right)=-e\varepsilon_{1}-\xi_{1}, \end{array}$$ (5)

$$\begin{array}{c} \min_{w_{2},b_{2}}\;\frac{1}{2}{\left\|Y+e\varepsilon_{2}-\left(Xw_{2}+eb_{2}\right)\right\|}_{2}^{2}+C_{2}{\xi_{2}}^{T}\xi_{2}\\ s.t.\;-Y+\left(Xw_{2}+eb_{2}\right)=-e\varepsilon_{2}-\xi_{2}, \end{array}$$ (6)

In formula (5) and (6), the square of L2-norm of slack variable $\xi_{1},\xi_{2}$ is used, instead of L1-norm in (2) and (3), which makes constraint $\xi_{1}\geq 0$ , $\xi_{2}\geq 0$ redundant, so the following formulas is obtained,

$$\min_{w_{1},b_{1}}\;\frac{1}{2}{\left\|Y-\left(Xw_{1}+eb_{1}\right)\right\|}_{2}^{2}+\frac{C_{1}}{2}\;{\left\|\left(Xw_{1}+eb_{1}\right)-Y-e\varepsilon_{1}\right\|}_{2}^{2},$$ (7)

$$\min_{w_{2},b_{2}}\;\frac{1}{2}{\left\|Y-\left(Xw_{2}+eb_{2}\right)\right\|}_{2}^{2}+\frac{C_{2}}{2}\;{\left\|-\left(Xw_{2}+eb_{2}\right)+Y-e\varepsilon_{2}\right\|}_{2}^{2},$$ (8)

(7) and (8) are two unconstrained QPPs, hence the solutions for w and b can be directly obtained by setting the derivatives to zero, as Eq. (9),

$$-H^{T}\left(\left(Y-\varepsilon_{1}e\right)-\text{Hu}\right)+c_{1}H^{T}\left(\text{Hu}-\left(Y-\varepsilon_{1}e\right)\right)=0,$$ (9)

where $u=\left[\begin{array}{c} w\\ b \end{array}\right]$ , $H=\left[X\;e\right]$ , then, we have Eq. (10) and Eq. (11),

$$u_{1}=\left[\begin{array}{c} w_{1}\\ b_{1} \end{array}\right]={\left(1+C_{1}\right)}^{2}{\left(H^{T}H\right)}^{-1}H^{T}Y+\left(1+C_{1}\right)C_{1}{\left(H^{T}H\right)}^{-1}H^{T}\varepsilon_{1}e,$$ (10)

$$u_{2}=\left[\begin{array}{c} w_{2}\\ b_{2} \end{array}\right]={\left(1+C_{2}\right)}^{2}{\left(H^{T}H\right)}^{-1}H^{T}Y-\left(1+C_{2}\right)C_{2}{\left(H^{T}H\right)}^{-1}H^{T}\varepsilon_{2}e,$$ (11)

thus, the final regression function is $f\left(X\right)=\frac{1}{2}X\left(w_{1}^{T}+w_{2}^{T}\right)+\frac{1}{2}\left(b_{l}+b_{2}\right)$ .

#### 2.2.2 Improved LSTSVR (ILSTSVR)

When applying LSTSVR to handle high-dimensional genotype datasets with small samples, the model is prone to a high risk of overfitting. This is due to the fact that the model may overly fit noise and feature details in the training set, leading to decrease generalization performance on new samples and thereby affecting the effectiveness and reliability of the predictive results. Therefore, this study adds a Lasso regularization term for the weight parameter w in the LSTSVR framework. For linear problems, the function of ILSTSVR is as follows,

$$\begin{array}{c} \min_{w_{1},b_{1}}\;\frac{1}{2}{\left\|Y-\varepsilon_{1}e-\left(Xw_{1}+b_{1}e\right)\right\|}_{2}^{2}+\frac{C_{1}}{2}{\xi_{1}}^{T}\xi_{1}+\frac{C_{3}}{2}\left({\left\|w_{1}\right\|}_{1}+b_{1}^{2}\right)\\ s.t.\;Y-\left(Xw_{1}+b_{1}e\right)=-\varepsilon_{1}e-\xi_{1} \end{array},$$ (12)

$$\begin{array}{c} \min_{w_{2},b_{2}}\;\frac{1}{2}{\left\|Y+\varepsilon_{2}e-\left(Xw_{2}+b_{2}e\right)\right\|}_{2}^{2}+\frac{C_{2}}{2}{\xi_{2}}^{T}\xi_{2}+\frac{C_{4}}{2}\left({\left\|w_{2}\right\|}_{1}+b_{2}^{2}\right)\\ s.t.\;-Y+\left(Xw_{2}+b_{2}e\right)=-\varepsilon_{2}e-\xi_{2} \end{array},$$ (13)

where $C_{1}$ , $C_{2}$ , $C_{3}$ , $C_{4}$ , $\varepsilon_{1}$ , $\varepsilon_{2}$ are positive penalty parameters, $\xi_{1},\xi_{2}$ are slack variables, e is a vector of ones of appropriate dimensions. For the non-differentiability with L1 regularization and the convenience of calculations, we assume ${\left\|w\right\|}_{1}={\left\|\alpha *w\right\|}_{2}^{2}$ , then (12) and (13) can be converted into as follow,

$$\begin{array}{c} \min_{w_{1},b_{1}}\;\frac{1}{2}{\left\|Y-\varepsilon_{1}e-\left(Xw_{1}+b_{1}e\right)\right\|}_{2}^{2}+\frac{C_{1}}{2}\;{\left\|\left(Xw_{1}+b_{1}e\right)-Y-\varepsilon_{1}e\right\|}_{2}^{2}\\ +\frac{C_{3}}{2}\left({\left\|{\alpha_{1}*w}_{1}\right\|}_{2}^{2}+b_{1}^{2}\right), \end{array}$$ (14)

$$\begin{array}{c} \min_{w_{2},b_{2}}\;\frac{1}{2}{\left\|Y+\varepsilon_{2}e-\left(Xw_{2}+b_{2}e\right)\right\|}_{2}^{2}+\frac{C_{2}}{2}\;{\left\|-\left(Xw_{2}+b_{2}e\right)+Y-\varepsilon_{2}e\right\|}_{2}^{2}\\ +\frac{C_{4}}{2}\left({\left\|{\alpha_{2}*w}_{2}\right\|}_{2}^{2}+b_{2}^{2}\right), \end{array}$$ (15)

where $\alpha_{1}$ , $\alpha_{2}$ are vectors of appropriate dimensions, * represents element-wise multiplication. Expand (14) yields,

$$\begin{array}{c} L=\min\;\frac{1}{2}{\left(Y-\varepsilon_{1}e-\left(Xw_{1}+b_{1}e\right)\right)}^{T}\left(Y-\varepsilon_{1}e-\left(Xw_{1}+b_{1}e\right)\right)\\ +\frac{C_{1}}{2}{\left(\left(Xw_{1}+b_{1}e\right)-Y-\varepsilon_{1}e\right)}^{T}\left(\left(Xw_{1}+b_{1}e\right)-Y-\varepsilon_{1}e\right)\\ +\frac{C_{3}}{2}{\left({diag\left(\alpha_{1}\right)w}_{1}\right)}^{T}\left({diag\left(\alpha_{1}\right)w}_{1}\right)+\frac{C_{3}}{2}b_{1}^{2}, \end{array}$$ (16)

let the derivatives of (16) with respect to $w_{1}$ and $b_{1}$ respectively be zero, we obtain Eq. (17) and Eq. (18),

$$\begin{array}{c} \frac{\partial L}{\partial w_{1}}=-X^{T}\left(Y-Xw_{1}-b_{1}e-\varepsilon_{1}e\right)+C_{1}X^{T}\left(Xw_{1}+b_{1}e-\varepsilon_{1}e-Y\right)\\ +C_{3}{diag\left(\alpha_{1}\right)}^{T}diag\left(\alpha_{1}\right)w_{1}=0, \end{array}$$ (17)

$$\begin{array}{c} \frac{\partial L}{\partial b_{1}}=-e^{T}\left(Y-Xw_{1}-b_{1}e-\varepsilon_{1}e\right)+C_{1}e^{T}\left(Xw_{1}+b_{1}e-\varepsilon_{1}e-Y\right)\\ +C_{3}b_{1}=0, \end{array}$$ (18)

composed in matrix form as Eq. (19),

$$\left[\begin{array}{cc} {\left(1+C_{1}\right)X}^{T}X+C_{3}D_{1} & {\left(1+C_{1}\right)X}^{T}e\\ \left(1+C_{1}\right)e^{T}X & \left(1+C_{1}\right)e^{T}e+C_{3} \end{array}\right]\left[\begin{array}{c} w_{1}\\ b_{1} \end{array}\right]=\left[\begin{array}{cc} \left(1+C_{1}\right)X^{T} & \left(C_{1}-1\right)X^{T}e\\ \left(1+C_{1}\right)e^{T} & \left(C_{1}-1\right)e^{T}e \end{array}\right]\left[\begin{array}{c} Y\\ \varepsilon_{1} \end{array}\right]$$ (19)

where $D_{1}={\text{diag}\left(\alpha_{1}\right)}^{T}\text{diag}\left(\alpha_{1}\right)$ . Similarly, the following result Eq. (20) can be obtained through (15),

$$\left[\begin{array}{cc} {\left(1+C_{2}\right)X}^{T}X+C_{4}D_{2} & {\left(1+C_{2}\right)X}^{T}e\\ \left(1+C_{2}\right)e^{T}X & \left(1+C_{2}\right)e^{T}e+C_{4} \end{array}\right]\left[\begin{array}{c} w_{2}\\ b_{2} \end{array}\right]=\left[\begin{array}{cc} \left(1+C_{2}\right)X^{T} & \left(1-C_{2}\right)X^{T}e\\ \left(1+C_{2}\right)e^{T} & \left({1-C}_{2}\right)e^{T}e \end{array}\right]\left[\begin{array}{c} Y\\ \varepsilon_{2} \end{array}\right]$$ (20)

where $D_{2}={\text{diag}\left(\alpha_{2}\right)}^{T}\text{diag}\left(\alpha_{2}\right)$ . Because of the assumption ${\left\|w\right\|}_{1}={\left\|\alpha *w\right\|}_{2}^{2}$ , we set an initial $\alpha^{0}$ , and calculate the final w and b through the iterative formula that updates alternately, as Eq. (21)

$${\alpha_{i}}^{k+1}=\sqrt{\frac{1}{\left|{w_{i}}^{k}\right|}}$$ (21)

To make the process clear, the computational process is summarized as Table 1.

**TABLE 1:** Iterative algorithm to solve the L1 regularization problem.

Algorithm 1: An iterative algorithm to solve the L1 regularization problem in (14) and (15)
**Input:** $\varepsilon_{1}>0$ **,** $\varepsilon_{2}>0$ **,** $C_{1}>0$ **,** $C_{2}>0$ **,** $C_{3}>0$ **,** $C_{4}>0$ **,** $X\in R^{n\times m}$ **,** $Y\in R^{n\times 1}$
**Output:** $w_{1}$ **,** $b_{1}$ and $w_{2}$ **,** $b_{2}$
1: Random initialization $\alpha_{1}^{0}$ **,** $\alpha_{2}^{0},$ set the number of iterations t = 0
2: **repeat**
Calculate and update $w_{1}$ **,** $b_{1}$ using (19)
Calculate and update $w_{2}$ **,** $b_{2}$ using (20)
Accordingto the formula ${\alpha_{i}}^{k+1}=\sqrt{\frac{1}{\left|{w_{i}}^{k}\right|}}$ ,item-by-item update of ${\;\alpha }_{1}$ **,** ${\;\alpha }_{2}$ t=t+1
**until** ${\;\alpha }_{1}$ **,** ${\;\alpha }_{2}$ *convergence*

Then we obtain Eq. (22),

$$f\left(X\right)=\frac{1}{2}X\left(w_{1}^{T}+w_{2}^{T}\right)+\frac{1}{2}\left(b_{l}+b_{2}\right)$$ (22)

For nonlinear problems, kernel functions typically provide a good solution, and here we have chosen RBF as the kernel function for our model. The formula of RBF as Eq. (23),

$$K\left(x,x^{\prime }\right)=\exp\left(-\frac{{\left\|x-x^{\prime }\right\|}^{2}}{2\sigma^{2}}\right),$$ (23)

where σ is kernel parameter, the value has a significant impact on prediction performance, easily leading to overfitting or underfitting.

We map the training data through $K\left(X,X^{T}\right)$ into a high-dimensional reproducing kernel Hilbert space (RKHS) (Aronszajn, 1950), obtaining matrix H. Thus, we obtain the function for the nonlinear problem as Eq. (24) and Eq. (25),

$$\begin{array}{c} \min_{w_{1},b_{1}}\;\frac{1}{2}{\left\|Y-\varepsilon_{1}e-\left(Hw_{1}+b_{1}e\right)\right\|}_{2}^{2}+\frac{C_{1}}{2}{\xi_{1}}^{T}\xi_{1}+\frac{C_{3}}{2}\left({\left\|w_{1}\right\|}_{1}+b_{1}^{2}\right)\\ s.t.\;Y-\left(Xw_{1}+b_{1}e\right)=-\varepsilon_{1}e-\xi_{1}, \end{array}$$ (24)

$$\begin{array}{c} \min_{w_{2},b_{2}}\;\frac{1}{2}{\left\|Y+\varepsilon_{2}e-\left(Hw_{2}+b_{2}e\right)\right\|}_{2}^{2}+\frac{C_{2}}{2}{\xi_{2}}^{T}\xi_{2}+\frac{C_{4}}{2}\left({\left\|w_{2}\right\|}_{1}+b_{2}^{2}\right)\\ s.t.\;-Y+\left(Xw_{2}+b_{2}e\right)=-\varepsilon_{2}e-\xi_{2}, \end{array}$$ (25)

Similarly, we can obtain Eq. (26) and Eq. (27),

$$\left[\begin{array}{cc} {\left(1+C_{1}\right)H}^{T}H+C_{3}D_{1} & {\left(1+C_{1}\right)H}^{T}e\\ \left(1+C_{1}\right)e^{T}H & \left(1+C_{1}\right)e^{T}e+C_{3} \end{array}\right]\left[\begin{array}{c} w_{1}\\ b_{1} \end{array}\right]=\left[\begin{array}{cc} \left(1+C_{1}\right)H^{T} & \left(C_{1}-1\right)H^{T}e\\ \left(1+C_{1}\right)e^{T} & \left(C_{1}-1\right)e^{T}e \end{array}\right]\left[\begin{array}{c} Y\\ \varepsilon_{1} \end{array}\right],$$ (26)

$$\left[\begin{array}{cc} {\left(1+C_{2}\right)H}^{T}H+C_{4}D_{2} & {\left(1+C_{2}\right)H}^{T}e\\ \left(1+C_{2}\right)e^{T}H & \left(1+C_{2}\right)e^{T}e+C_{4} \end{array}\right]\left[\begin{array}{c} w_{2}\\ b_{2} \end{array}\right]=\left[\begin{array}{cc} \left(1+C_{2}\right)H^{T} & \left(1-C_{2}\right)H^{T}e\\ \left(1+C_{2}\right)e^{T} & \left({1-C}_{2}\right)e^{T}e \end{array}\right]\left[\begin{array}{c} Y\\ \varepsilon_{2} \end{array}\right],$$ (27)

the final nonlinear model as Eq. (28),

$$f\left(H\right)=\frac{1}{2}H\left(w_{1}^{T}+w_{2}^{T}\right)+\frac{1}{2}\left(b_{l}+b_{2}\right)$$ (28)

#### 2.2.3 Optimizing ILSTSVR using SABO

For the non-linear ILSTSVR model, the choice of kernel parameter σ for RBF has a significant impact on prediction performance. Moreover, in this study, we set $C_{1}=C_{2}$ and $C_{3}=C_{4}$ for the ILSTSVR parameters $C_{1}$ , $C_{2}$ , $C_{3}$ , $C_{4}$ as given in (12) and (13). Parameter optimization plays an important role in machine learning, as selecting appropriate parameters can significantly enhance a model’s predictive capability and accuracy. Different combinations of parameters may lead to vastly different performances of the model on both training and testing data. Furthermore, parameters affect the model complexity and learning capacity. By adjusting them, we can better strike a balance between overfitting and underfitting (Young et al., 2015). Although grid search can find the global optimal solution, it will result in enormous computational resource consumption and neglect the correlations among parameters (Vincent and Jidesh, 2023). Instead, we use the recently proposed SABO for parameter optimization, which updates the positions of population members in the search space using subtraction averages of individuals, characterized by strong optimization capability and fast convergence rates (Moustafa et al., 2023).

The basic inspiration for the design of the SABO is mathematical concepts such as averages, the differences in the positions of the search agents, and the sign of difference of the two values of the objective function. The idea of using the arithmetic mean location of all the search agents (i.e., the population members of the *t*th iteration), instead of just using, e.g., the location of the best or worst search agent to update the position of all the search agents (i.e., the construction of all the population members of the (*t* + 1)th iteration), is not new, but the SABO’s concept of the computation of the arithmetic mean is wholly unique, as it is based on a special operation " ${-}_{v}$ ", called the v-subtraction of the search agents B from the search agent A, which is defined as Eq. (29):

$$A\;{-}_{v}\;B=sign\left(F\left(A\right)-F\left(B\right)\right)\left(A-\vec{v}*B\right),$$ (29)

where $\vec{v}$ is a vector of the dimension m, the operation "∗" represents the Hadamard product of the two vectors, F(A) and F(B) are the values of the objective function of the search agents A and B, respectively, and sign is the signum function (Trojovský and Dehghani, 2023).

In the proposed SABO, the displacement of any search agent $X_{i}$ in the search space is calculated by the arithmetic mean of the *v*-subtraction of each search agent $X_{j}$ , j = 1,2, … , *N*, from the search agent $X_{i}$ . Thus, the new position for each search agent is calculated using (30).

$$X_{i}^{new}=X_{i}+{\vec{r}}_{i}*\frac{1}{N}\sum_{j=1}^{N}\left(X_{i}\;{-}_{v}\;X_{j}\right),i=1,2,\ldots ,N,$$ (30)

where $X_{i}^{\text{new}}$ is the new proposed position for the *i*th search agent $X_{i}$ , N is the total number of the search agents, and ${\vec{r}}_{i}$ is a vector of the dimension m. Then, if this proposed new position leads to an improvement in the value of the objective function, it is acceptable as the new position of the corresponding agent, according to (31)

$$X_{i}=\left\{\begin{array}{l} X_{i}^{new},F_{i}^{new}<F_{i};\\ X_{i},else, \end{array}\right.$$ (31)

where $F_{i}$ and $F_{i}^{\text{new}}$ are the fitness function values of the search agents $X_{i}$ and $X_{i}^{\text{new}}$ , respectively.

Similar to other optimization algorithms, the primary positions of the search agents in the search space are randomly initialized using (32).

$$x_{i,d}=lb_{d}+r_{i,d}∙\left(ub_{d}-lb_{d}\right),i=1,\ldots ,N,d=1,\ldots ,m,$$ (32)

where $x_{i,d}$ is the *d*th dimension of $X_{i}$ , N is the number of search agents, m is the number of decision variables, $r_{i,d}$ is a random number in the interval [0, 1], and $lb_{d}$ and $ub_{d}$ are the lower and upper bounds of the *d*th decision variables, respectively (Trojovský and Dehghani, 2023).

Here, we define the fitness function for SABO as MSE function shown in (34), $C_{1}\in \left[C_{1_{\min}},C_{1_{\max}}\;\right]$ , $C_{3}\in \left[C_{3_{\min}},C_{3_{\max}}\;\right]$ and $\sigma \in \left[\sigma_{\min},\sigma_{\max}\right]$ . The parameter corresponding to the smallest fitness function value obtained through iteration is the optimal combination of parameters for ILSTSVR. Finally, train the model according to the obtained parameters combination.

#### 2.2.4 Performance evaluation

To evaluate the prediction performance of GEBVs by the model, while avoiding the problem that Pearson correlation coefficient fails to measure the distance between true and predicted values, we adopt both Pearson correlation coefficient and MSE as evaluation metrics for the relationship between predicted and true values. Furthermore, we use ten-fold cross-validation to assess the model’s performance. The original dataset is divided into ten equally-sized (or nearly equal) folds; for each fold, it serves as the validation set, while the remaining nine folds constitute the training set. Training a model using the training set, and its performance is evaluated using the validation set. After completing all ten iterations, the average of the performance measures obtained from each validation set is taken, thereby yielding an overall assessment of the model’s performance.

The Pearson correlation coefficient (PCC) is used to measure the strength and direction of the linear relationship between two continuous variables, and is defined as follows,

$$\rho_{y,y^{\prime }}=\frac{cov\left(y,y^{\prime }\right)}{\sigma_{y}\sigma_{y^{\prime }}}$$ (33)

where y and $y^{\prime }$ represent the true values and predicted values respectively, $\text{cov}\left(y,y^{\prime }\right)$ denotes the covariance of vectors y and $y^{\prime }$ , $\sigma_{y}$ and $\sigma_{y^{\prime }}$ are the standard deviations of vector y and $y^{\prime }$ respectively.

Mean squared error (MSE) measures the degree of difference between predicted values and actual values, and is defined as follows,

$$MSE\left(y,y^{\prime }\right)=\frac{1}{n}\sum_{i=1}^{n}{\left(y_{i}-y_{i}^{\prime }\right)}^{2}$$ (34)

## 3 Result

### 3.1 Comparison of SABO-ILSTSVR with the base model

**FIGURE 2:**
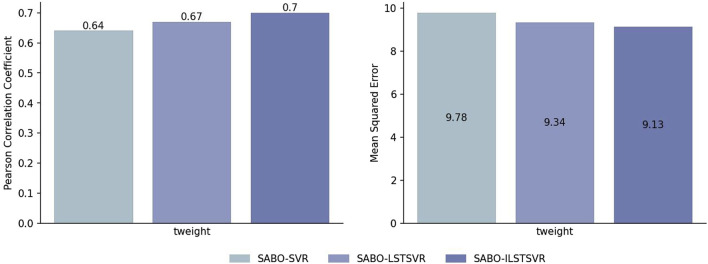
Performance of Pearson correlation coefficient and MSE prediction for SABO-SVR, SABO-LSTSVR, and SABO-ILSTSVR on the potato dataset.

To validate the effectiveness of ILSTSVR, this section compares SABO-ILSTSVR with some methods prior to its improvement on four datasets (potato, wheat, maize and brassica napus). The results on the potato dataset (Figure 2) show that the SABO-ILSTSVR exhibits a 4% increase in Pearson correlation coefficient and a 2% decrease in MSE compared with SABO-LSTSVR. Furthermore, when contrasted with SABO-SVR, the SABO-ILSTSVR demonstrates a 9% improvement in the Pearson correlation coefficient and a 6% decrease in MSE.

The results on other datasets are shown in Table 2. On the wheat dataset, SABO-ILSTSVR improves the Pearson correlation coefficient in four environments by an average of 2%, 2%, 8%, and 2% and reduces the MSE in four environments by an average of 1%, 3%, 1% and 1%, respectively, compared with SABO-SVR and SABO-LSTSVR. On the maize dataset, SABO-ILSTSVR improves the Pearson correlation coefficient by an average of 6% and reduces the MSE by 1%, respectively, compared with SABO-SVR and SABO-LSTSVR. On the brassica napus dataset, SABO-ILSTSVR improves the Pearson correlation coefficient in three traits by an average of 11%, 6%, and 24% and reduces the MSE in three traits by an average of 17%, 1% and 12%, respectively, compared with SABO-SVR and SABO-LSTSVR.

**TABLE 2:** Prediction performance in ten-fold cross-validation for each trait in wheat, maize and brassica napus datasets.

	SABO-SVR		SABO-LSTSVR		SABO-ILSTSVR	
Trait	PCC	MSE	PCC	MSE	PCC	MSE
env1	0.58	0.66	0.58	0.67	0.59	0.65
env2	0.5	0.76	0.49	0.75	0.51	0.73
env3	0.43	0.82	0.42	0.82	0.45	0.81
env4	0.52	0.71	0.5	0.76	0.52	0.73
yield	0.37	0.73	0.38	0.73	0.4	0.72
flower0	0.64	0.045	0.61	0.047	0.7	0.038
flower4	0.68	0.045	0.66	0.04	0.71	0.042
flower8	0.36	0.017	0.38	0.02	0.46	0.016

### 3.2 Comparison of SABO-ILSTSVR with other methods

Considering the complexity of the genetic architecture, this study employs real data to evaluate the prediction performance of models, including datasets from public available sources for maize, wheat, potato, and Brassica napus. And we performed standardization on the phenotype data of all datasets. Due to the small sample sizes in the adopted datasets, random sampling errors may be relatively substantial, leading to decreased model prediction accuracy, reduced statistical power of tests, and difficulty in obtaining stable and reliable statistical inferences (Bengio and Grandvalet, 2004). Consequently, a ten-fold cross-validation is applied to each dataset in this study, with the average of the results over ten iterations used to represent the ultimate prediction performance of the models.

This section compares the prediction performance of LightGBM, rrBLUP, GBLUP, BSLMM, BayesRR, Lasso, RF, SVR, DNNGP, and SABO-ILSTSVR across four datasets (potato, wheat, maize and brassica napus). The LightGBM model originates from a LightGBM Python package developed by Microsoft. The BayesRR, Lasso, RF, and SVR models are included in the Scikit-learn python library. The GBLUP, BSLMM and rrBLUP models utilize the sommer R package (Covarrubias-Pazaran, 2016), hibayes R package (Yin et al., 2022) and the rrBLUP R package (Endelman, 2011), respectively. The DNNGP model is mentioned in the paper by (Kelin et al., 2023).

#### 3.2.1 Potato dataset

**FIGURE 3:**
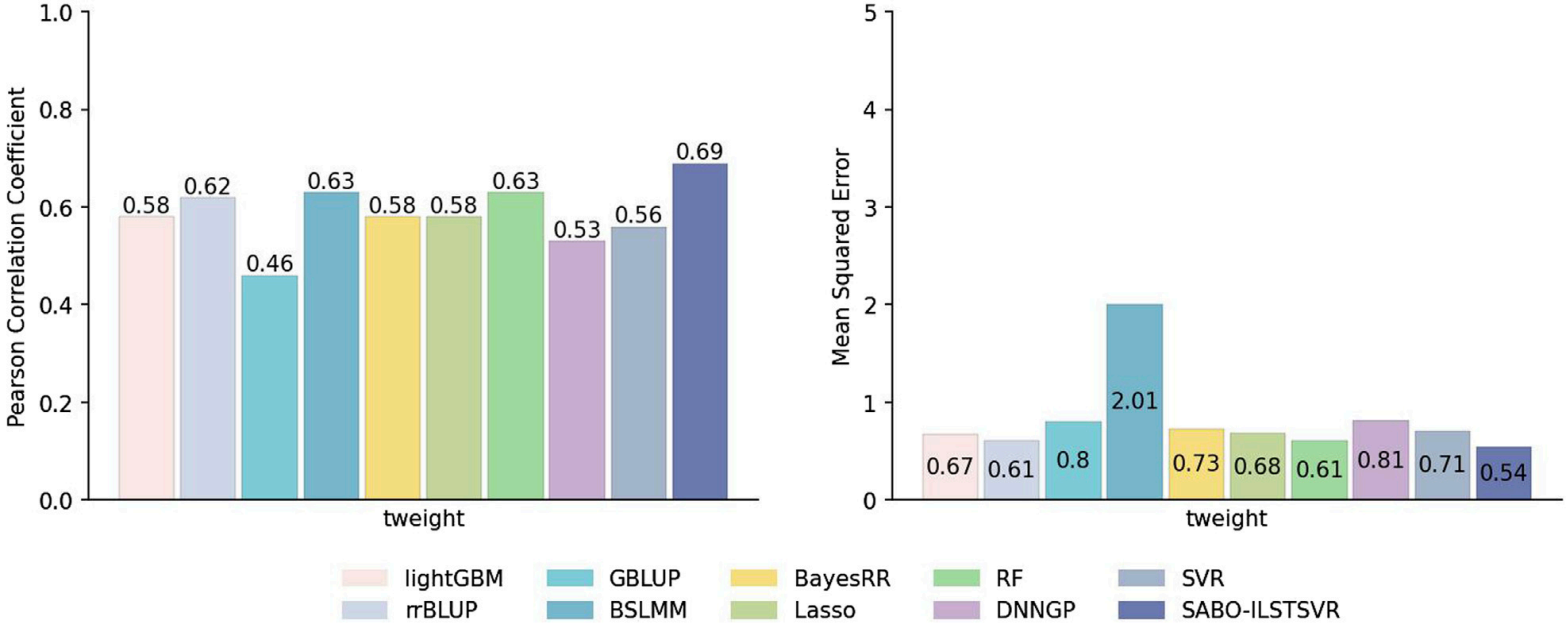
Performance of prediction for various models on the potato dataset in terms of Pearson correlation coefficients and MSE.

This paper first compares the prediction performance of SABO-ILSTSVR with LightGBM, rrBLUP, GBLUP, BSLMM, BayesRR, Lasso, RF, SVR, DNNGP on the potato dataset. Detailed information about SNPs and phenotypes in the potato dataset has been described in the Materials and Methods section. As shown in Figure 3, SABO-ILSTSVR outperforms comparative models across key performance metrics. Specifically, SABO-ILSTSVR improves the Pearson correlation coefficient by 18%, 11%, 50%, 9%, 18%, 18%, 9%, 23% and 30% and reduces the MSE by an average of 19%, 11%, 32%, 73%, 26%, 20%, 11%, 23% and 33%, respectively, compared with LightGBM, rrBLUP, GBLUP, BSLMM, BayesRR, Lasso, RF, SVR, DNNGP.

#### 3.2.2 Wheat dataset

**FIGURE 4:**
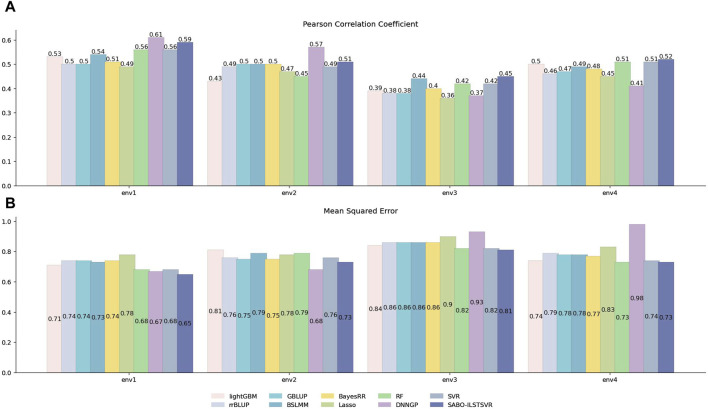
Performance of prediction for various models on the wheat dataset. Pearson correlation coefficient **(A)** and MSE **(B)** metrics of four phenotypes (env1, env2, env3 and env4), as evaluated through ten-fold cross-validation.

Similarly, prediction performance comparisons were conducted for SABO-ILSTSVR, LightGBM, rrBLUP, GBLUP, BSLMM, BayesRR, Lasso, RF, SVR, DNNGP on the wheat dataset. As shown in Figure 4, the DNNGP model exhibits the highest Pearson correlation coefficients in predicting yield under environments env1 and env2 of the wheat dataset, whereas its performance in env3 and env4 is inferior to that of other models. In contrast, our proposed SABO-ILSTSVR model demonstrates higher Pearson correlation coefficients and lower mean squared errors for yield data across all four environments compared with the other models. Specifically, SABO-ILSTSVR achieves the best performance in env3 and env4 and is second only to DNNGP in env1 and env2. The reason for the differing performance may be due to their prediction performance varying across different agroclimatic regions.

#### 3.2.3 Maize dataset

**FIGURE 5:**
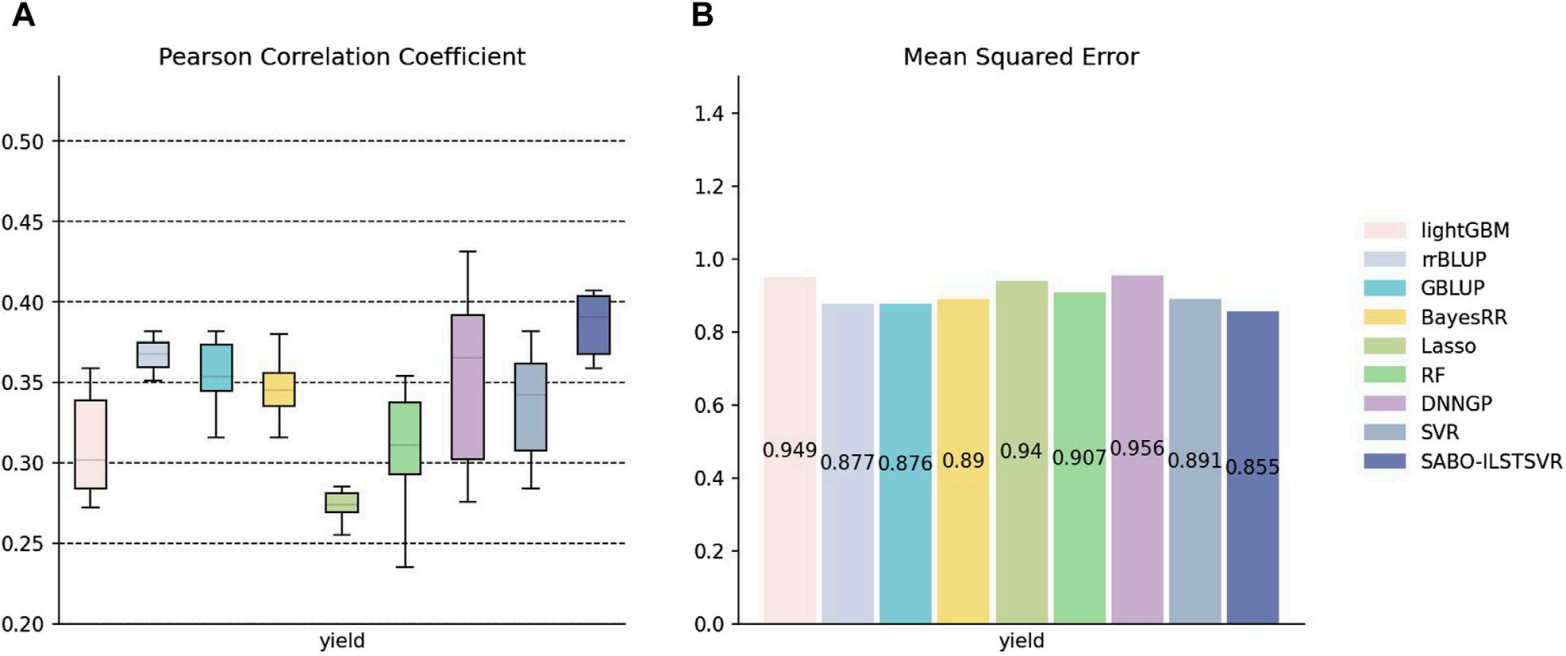
Performance of prediction for various models on the maize dataset. **(A)** Pearson correlation coefficients of maize yield traits, represented by box plots, after ten replicates ten-fold cross-validation. **(B)** Average MSE of maize yield traits after ten replicates ten-fold cross-validation.

The detailed information on SNPs and phenotypes in the maize dataset has been described in the Materials and Methods section. Unlike the potato and wheat datasets, this section performs ten replicates ten-fold cross-validation separately for SABO-ILSTSVR, LightGBM, rrBLUP, GBLUP, BayesRR, Lasso, RF, SVR, DNNGP on the maize dataset. Comparison was not conducted with BSLMM due to its unstable results. The Pearson correlation coefficients of the ten results are represented (A) in Figure 5, while the mean squared errors (MSEs) of the ten results are averaged and depicted as (B). As shown in Figure 5, Lasso exhibits the lowest prediction performance, whereas DNNGP, despite having the highest Pearson correlation coefficient prediction performance, displays significantly large variations across the ten runs, resulting in elongated bars in the boxplot. In contrast, SABO-ILSTSVR has a stable Pearson correlation coefficient prediction performance and exhibits the lowest MSE.

#### 3.2.4 Brassica napus dataset

**FIGURE 6:**
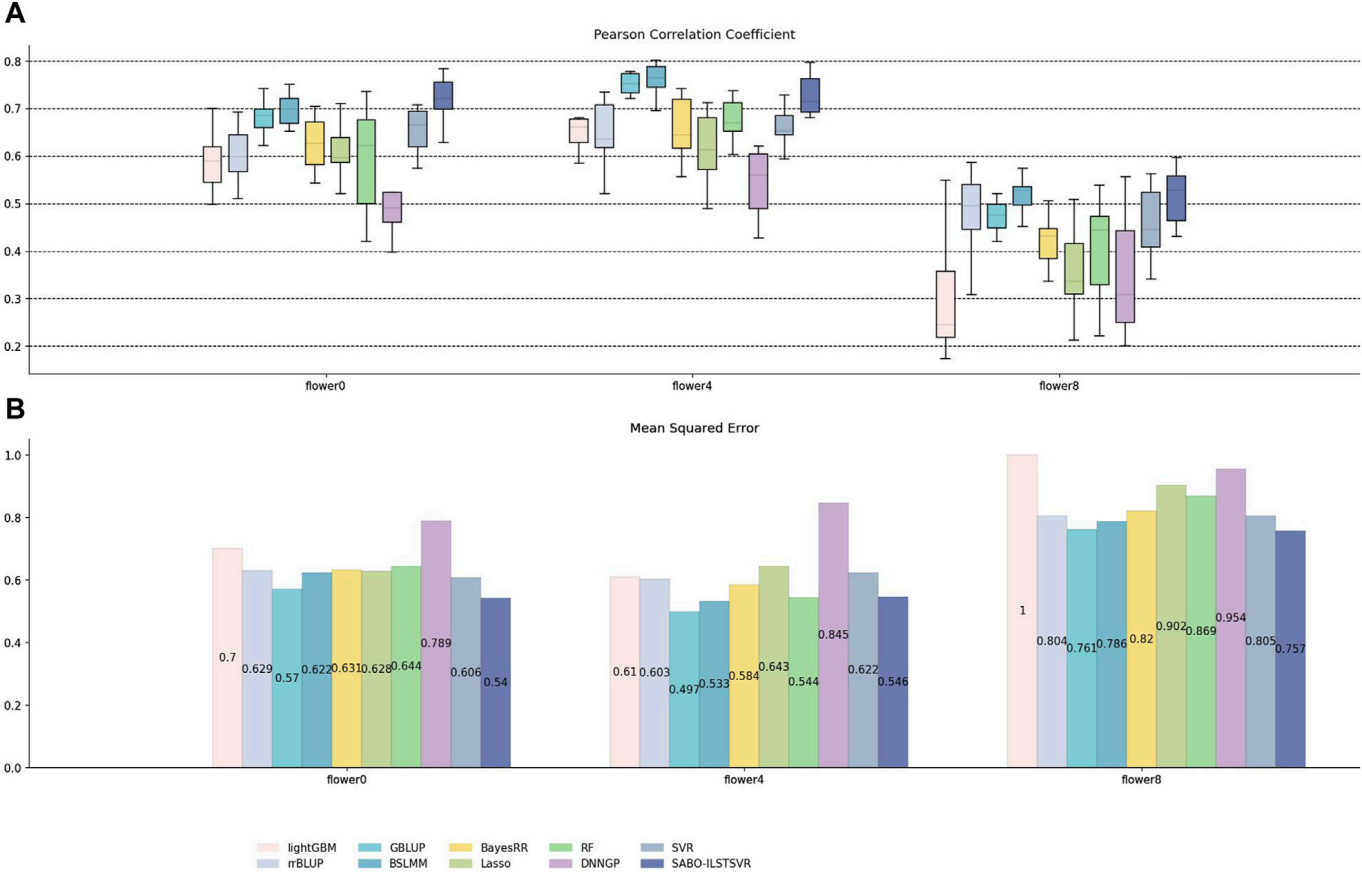
Performance of prediction for various models on the brassica napus dataset. **(A)** Pearson correlation coefficients of three phenotypes (flower0, flower4 and flower8), represented by box plots, after ten replicates ten-fold cross-validation. **(B)** Average MSE of three phenotypes after ten replicates ten-fold cross-validation.

Similarly, on the brassica napus dataset, ten replicates ten-fold cross-validation was performed for SABO-ILSTSVR, LightGBM, rrBLUP, GBLUP, BSLMM, BayesRR, Lasso, RF, SVR, DNNGP, respectively. The Pearson correlation coefficients of the ten results are represented by (A) in Figure 6, while the mean squared errors (MSEs) after averaging are depicted by (B). As shown in (A) of Figure 6, SABO-ILSTSVR exhibits the highest Pearson correlation coefficient and lowest MSE prediction performance on flower0 and flower8, whereas BSLMM achieves the highest Pearson correlation coefficient prediction performance on flower4. The prediction performance of the SABO-ILSTSVR model is slightly lower than that of the GBLUP and BSLMM model but higher than that of the other comparative models on flower4.

## 4 Discussion

In this study, we integrate Least Squares Twin Support Vector Regression (LSTSVR) with Lasso regularization, constructing a GP model named ILSTSVR. We use the SABO optimization algorithm to effectively optimize the parameters of the model. To address the number of genotype samples is far less than the number of SNPs markers, we introduce a Lasso regularization term into LSTSVR. By the unique feature selection property of Lasso, it can effectively shrink the coefficients of non-key features to zero, achieving parameter sparsity and effectively preventing overfitting. Meanwhile, to cope with the potential nonlinear relationships in genotype data, we adopt the radial basis function (RBF) kernel, mapping the raw data into a high-dimensional space to attain linear separability.

Considering the differences in genotype data among various species may lead to distinct optimal parameters, we used the SABO optimization algorithm to automatically tune the parameters of the ILSTSVR model. To validate the effectiveness of this model, we conducted evaluations on multiple datasets spanning potato, maize, wheat, and Brassica napus, and compared its prediction performance against a series of widely-used models such as LightGBM, rrBLUP, GBLUP, BSLMM, BayesRR, Lasso, RF, SVR, DNNGP.

The results showed that the SABO-ILSTSVR model demonstrated outstanding prediction performance on the potato dataset, outperforming other benchmark models. In the wheat and brassica napus datasets containing multiple phenotypic traits, our model consistently exhibited higher prediction accuracy for most traits compared with other models. The box plot analysis of the maize and brassica napus dataset further revealed the robustness of the SABO-ILSTSVR model’s predictions.

In our further exploration of the future, confronted with the high-dimensional challenges of genomic data, we will delve deeper into how to efficiently perform feature extraction (Burges, 2009). With the continuous decline in sequencing costs, large-scale genomic sequencing of samples is poised to become a reality, the increased sample size may offer more favorable application for DL models (Khan et al., 2020; Yang et al., 2024). We will investigate innovative DL models within the field of breeding.

## Data Availability

The original contributions presented in the study are included in the article/Supplementary Material, further inquiries can be directed to the corresponding author.
